# Metagenomics as a Tool To Monitor Reclaimed-Water Quality

**DOI:** 10.1128/AEM.00724-20

**Published:** 2020-08-03

**Authors:** Pei-Ying Hong, David Mantilla-Calderon, Changzhi Wang

**Affiliations:** a Water Desalination and Reuse Center, Division of Biological and Environmental Science and Engineering, King Abdullah University of Science and Technology, Thuwal, Saudi Arabia; University of Illinois at Urbana-Champaign

**Keywords:** metagenomics, reclaimed water, water quality

## Abstract

Many biological contaminants are disseminated through water, and their occurrence has potential detrimental impacts on public and environmental health. Conventional monitoring tools rely on cultivation and are not robust in addressing modern water quality concerns. This review proposes metagenomics as a means to provide a rapid, nontargeted assessment of biological contaminants in water. When further coupled with appropriate methods (e.g., quantitative PCR and flow cytometry) and bioinformatic tools, metagenomics can provide information concerning both the abundance and diversity of biological contaminants in reclaimed waters. Further correlation between the metagenomic-derived data of selected contaminants and the measurable parameters of water quality can also aid in devising strategies to alleviate undesirable water quality. Here, we review metagenomic approaches (i.e., both sequencing platforms and bioinformatic tools) and studies that demonstrated their use for reclaimed-water quality monitoring. We also provide recommendations on areas of improvement that will allow metagenomics to significantly impact how the water industry performs reclaimed-water quality monitoring in the future.

## Abstract (precis)

Many biological contaminants are disseminated through water, and their occurrence has potential detrimental impacts on public and environmental health. Conventional monitoring tools rely on cultivation and are not robust in addressing modern water quality concerns. This review proposes metagenomics as a means to provide a rapid, nontargeted assessment of biological contaminants in water. When further coupled with appropriate methods (e.g., quantitative PCR and flow cytometry) and bioinformatic tools, metagenomics can provide information concerning both the abundance and diversity of biological contaminants in reclaimed waters.

## INTRODUCTION

Water scarcity in the Middle East and North Africa regions as well as in countries such as Singapore, Australia, and the Maldives has necessitated the use of reclaimed water to alleviate the depletion of nonrenewable freshwater supplies. Reclaimed water is increasingly used in landscape irrigation to maintain green living spaces and for agricultural irrigation to produce food. Reclaimed water is also injected into aquifers to replenish depleting groundwater and used as an energy exchange medium in cooling towers. In some places, reclaimed water further undergoes advanced treatment processes, typically involving reverse-osmosis membrane filtration, to become a potable water source. Depending on the intended reuse purpose, different wastewater treatment technologies are used to provide the reclaimed water with quality that abides by either World Health Organization (WHO) guidelines or standards inspired by the U.S. Environmental Protection Agency (EPA) and the International Organization for Standardization (ISO).

Current regulations stipulated by the WHO, U.S. EPA, and ISO require only the enumeration of fecal indicators (e.g., total and fecal coliforms) to indicate reclaimed-water quality. The standard methods used to determine these fecal indicators can be prone to false-negative results if viable bacteria are stressed or injured. Culture-based methods also require time (typically 24 h to 48 h) for the microbial targets to grow to levels that facilitate enumeration. This process impedes the ability for a rapid response. Furthermore, fecal indicators do not occur at frequencies that correlate well with waterborne pathogens in reclaimed water (1); hence, they cannot predict accurately the presence of pathogens. Considering these limitations, standard methods have become increasingly obsolete in addressing modern water quality concerns, especially because emerging contaminants are found in reclaimed waters intended for agriculture and landscape irrigation and can potentially affect public health. These contaminants include bacterial pathogens (particularly those related to antibiotic-resistant ones), viral pathogens, protozoal hosts for intracellular pathogens, and extracellular DNA (e.g., antibiotic resistance genes [ARGs]) (2, 3). Many of these pathogens are fastidious, slow growing, and difficult to culture for routine monitoring.

Besides culture-based approaches, molecular methods such as quantitative PCR (qPCR) can determine the presence of pathogens or antibiotic resistance genes. However, qPCR is a targeted approach that detects only the marker genes that hybridize to the designed primers or probes. Hence, this targeted approach would not provide insights into unknown gene targets that do not have any available primer sets. Given the wide spectrum of contaminants that are present, nontargeted methods that can provide information on both the phylogenetic and functional diversities of emerging contaminants simultaneously would be preferred. Additionally, the method should preferably provide quantitative estimates of those targets of interest to facilitate evidence-based decision-making. Some of the key questions to be asked when evaluating reclaimed water quality include the following. Is the wastewater treatment system functioning well to provide reclaimed water of the required quality? Is the reclaimed water biologically stable, and would it not change much in its quality along the distribution network? Are contaminants present in the reclaimed water that would affect the environment and consumers’ health at the point of use? Can we infer the presence of nutrients or chemical contaminants in the reclaimed water based on the presence of some of the microbial contaminants?

In this minireview, we argue that metagenomics is suitable to address the above-mentioned questions, hence facilitating reclaimed-water quality monitoring. We derived this proposition based on the following evidence gathered from the current literature: (i) advances in sequencing technologies have rapidly decreased the associated costs while increasing the number of raw reads available, (ii) the availability of bioinformatic tools to facilitate the analysis of metagenomic data allows the collection of massive data sets that reveal gene and functional diversities in a nontargeted manner, and (iii) the continuous improvement of both sequencing technologies and analytical tools is shortening the time required to perform metagenomics and analysis. However, its ability to provide quantitative measurements, good accuracy, and fine resolution of phylogenetic and functional classifications in mixed-community samples will need to be further improved to fully address the needs of reclaimed-water quality monitoring.

(This review was written based on the content presented by P.-Y. Hong at the 2018 Singapore International Water Week.)

## 

### Definition of metagenomics.

**FIG 1:**
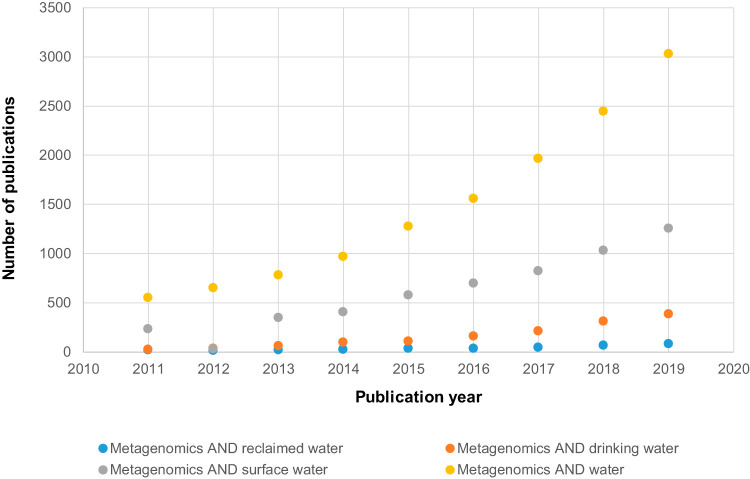
Number of publications associated with each keyword set and retrieved from Scopus from 2011 to 2019.

Metagenomics, being DNA based, can provide information on only who is there (i.e., taxonomic and phylogenetic information) and what is there (i.e., functional gene diversity). Depending on the type of microbial target (i.e., viruses, bacteria, or protozoa), different sample preparation measures and extraction protocols would have to be used to maximize the yield of DNA from these microorganisms before metagenomics is performed. However, because the microbial populations and genes detected by metagenomics are derived from DNA, they may be from nonviable cells or genes that are not being expressed. This approach contrasts with metatranscriptomics (RNA-based sequencing) or metaproteomics (peptide sequencing), which provides information on which microbial populations are alive, actively transcribing their genes, and translating the mRNA into proteins. Metagenomics should not be confused with amplicon-based high-throughput sequencing, which typically involves only a targeted gene (e.g., 16S rRNA or 18S rRNA genes) (4–6). Metagenomics should not be confused with whole-genome sequencing, which refers to single-genome sequencing. The number of papers related to the keywords “metagenomics” and various types of water matrices demonstrates that metagenomics is more widely utilized for surface waters than for reclaimed water (Fig. 1). However, the number of papers related to the use of metagenomics also experienced a high rate of increment, particularly from the year 2013 onward, with the advent and accessibility of sequencing technologies.

### Functional metagenomics.

Before metagenomics became more mainstream, an earlier approach involved extracting a large amount (e.g., >10 μg) of high-molecular-weight DNA from a sample (7), creating DNA fragments using endonucleases, and then ligating these DNA fragments into artificial chromosome vectors. The size of these DNA fragments can vary from a few kilobases to as long as more than 10 kb, depending on the fragment size that can be efficiently inserted into the vector. For instance, phage vectors accept inserts of approximately 15 to 20 kb, while those of bacterial artificial chromosomes can range from 150 to 350 kbp (8, 9). After gene insertion, the vectors are transformed into Escherichia coli, and individual transformants are expressed and screened for the intended functional traits. Transformants that express the intended functional traits are then sequenced to denote the identities of the inserted genes. Alternatively, all the transformants can be pooled and sequenced directly without any prescreening. The depth of information derived from this approach of functional metagenomics is limited by the number of transformants picked for screening and sequencing, but this limitation can be easily resolved using an automated colony picker. However, because it involves cloning and incubating cells before sequencing, this approach is subject to additional bias during cloning and takes a longer time for completion. Due to the amount of time and effort required, functional metagenomics does not facilitate efficient decision-making; hence, it has not been widely used for reclaimed-water quality monitoring.

However, the advantage of functional metagenomics is that inserted genes can express their enzymes, and subsequent biochemical characterization of these enzymes may lead to useful products. For example, Song et al. extracted high-molecular-weight DNA from the contents of the rumen and fragmented these DNAs to sizes ranging from 10 to 50 kbp before creating fosmid libraries. The clones were screened for cellulolytic activity, and those with positive cellulolytic activity were pooled for DNA extraction and sequencing (10). Further gene annotation revealed a novel glycosyl hydrolase family 5 cellulase gene with endo-β-1,4-glucanase. Although not demonstrated in that study, because of its potential application, this approach can potentially result in enzymes that can be applied to disrupt undesirable biofilms (11, 12).

### Current sequencing platforms for modern metagenomic approaches.

In daily routine monitoring of reclaimed-water quality, utilities may assess the biological stability of their reclaimed water. Biological stability is defined as the steady-state concentration of bacterial cells and composition in the water (13). A sudden increase in the concentration of bacterial cells would infer either the growth of microorganisms, an influx of microbial contaminants, or a failing distribution network, which might detrimentally impact operations and safety at the point of use. In addition to monitoring for biologically stable reclaimed water, utilities may also be interested in determining the performances of their treatment processes by tracking log removal values. This can be done by enumerating the concentrations of contaminants before and after treatment. Furthermore, to determine the risks associated with pathogens in our reclaimed-water supplies, quantitative estimates of pathogens are needed to facilitate microbial risk assessments. These questions require a timely response and modern metagenomic approaches (also referred to as shotgun sequencing), bypassing the need for cloning and cultivation and showing promise to address these questions.

A succession of sequencing platforms is available, from the now-defunct 454 pyrosequencing and Ion Torrent platforms to the current mainstream Illumina platform, along with the Nanopore and PacBio platforms, which can generate longer reads than Illumina reading chemistries depending on the quality and fragment size of the DNA template. Regardless of the sequencing platform, the main distinguishing feature is the ability to generate a large number of short reads (typically 100 to 300 bp per read) per run (Table 1) at costs typically ranging from $1,000 to $3,000 per run. Most of these sequencing platforms require significantly lower concentrations of DNA (typically 10 ng to 1 μg of DNA) than the clone-based functional metagenomic approach. The DNA amount required is small because modern sequencing platforms rely on solid-phase or emulsion-based PCR to exponentially amplify the gene molecules so that the detection sensitivity can be enhanced. However, this can also introduce amplification bias incurred during PCR (14) and sequencing errors due to low-fidelity polymerase (15). Shotgun sequencing also does not require DNA to be of a high molecular weight because the library preparation steps require DNA to be fragmented to approximately 400 bp before ligation with the index adaptors. However, overly fragmenting DNA will also impair the sequencing quality by generating reads with lengths shorter than the norm. Therefore, the optimization of protocols is required to minimize associated error rates and lapses in sequencing quality.

**TABLE 1:** Current sequencing platforms and their average read lengths and throughputs reported by either manufacturers or selected service laboratories

Platform	Directional read type	Read length (bp)	Throughput per lane	Reference
NovaSeq 6000				
SP flow cell	Single reads	100	400 million–500 million	63
	Paired reads	2 × 150 or 2 × 250	800 million	
S1 flow cell	Single reads	100	800 million	
	Paired reads	2 × 100 or 2 × 150	1.5 billion	
S2 flow cell	Single reads	100	1.5 billion	
S4 flow cell	Paired reads	2 × 150	5 billion–6 billion	

HiSeq 4000				
8-lane flow cell	Single reads	50–150	300 million–400 million	63
	Paired reads	50–150	650 million–800 million	

HiSeq 2500				
Rapid V2 flow cell	Single reads	50–260	150 million–200 million	63
	Paired reads	50–260	220 million–400 million	

MiSeq				
V3 flow cell	Paired reads	300	10 million–30 million	63
V2 flow cell	Paired reads	250	6 million–20 million	
V2 nano flow cell	Paired reads	250	500,000–2 million	

Flongle	Single reads	Dependent on the quality and fragment size of the DNA template	2 Gbp	64
MinION Mk and GridION Mk	Single reads	Dependent on the quality and fragment size of the DNA template	50 Gbp	64
PromethION	Single reads	Dependent on the quality and fragment size of the DNA template	220 Gbp	64
Sequel	Single reads	Dependent on the quality and fragment size of the DNA template but reportedly >1,000	500,000	65
Sequel II	Single reads	Dependent on the quality and fragment size of the DNA template but reportedly >1,000	4 million	65

### Availability of bioinformatic tools: genome-centric approach.

Sequencing results can be analyzed using either a genome-centric or a gene-centric approach. A genome-centric approach relies on assembling the short reads into contigs or scaffolds (larger genomic fragments) and further assembling the contigs or scaffolds into draft or complete genomes. Assembly can be performed with supervision, whereby reads are aligned against reference genomes based on sequence similarity. Homologous regions of the individual raw reads are also matched and linked together to form contigs in a *de novo* manner and are then aligned against reference genomes. Alternatively, assembly can be performed using an unsupervised approach that relies on discriminative sequence composition and/or coabundance of reads (16). The unsupervised approach groups contigs into bin clusters that are further differentiated based on the sequencing coverage. Contigs associated with a particular bin cluster can be retrieved for further *de novo* assembly to form draft population genomes. Several programs, including MetaBat (17), Concoct (18), and MaxBin (19), facilitate the reconstruction of microbial genomes from a metagenomic data set. The quality of the genome bins is further assessed using CheckM (20) to derive the percentage of completeness and the contamination level. For example, most draft genomes obtained via the unsupervised approach are classified as being of acceptable quality based on a substantial level of completeness (≥70%) and a low level of contamination (≤5%) (20, 21).

A genome-centric approach can potentially be used to identify the presence of pathogens in reclaimed water although not without challenge. Assuming that typical reclaimed water may have up to 2,000 unique species with an average genome size of 4 Mbp (22), each in equal relative abundances, 8 Gbp of reads would have to be obtained per sample to achieve 1× sequencing coverage of all genomes in this sample. An ecosystem with an equal distribution of species is unlikely, and a higher likelihood of assembling a genome usually applies to microbial cells that are predominant and, hence, overrepresented in terms of sequencing reads. This phenomenon does not consider that the current sequencing platforms require PCR to amplify gene targets before sequencing, thereby incurring a selective bias against those with a GC-rich genome (and, hence, achieving lower sequencing coverage). In most instances, trying to identify a unique genome confidently requires more than 5× sequencing coverage (23). Even higher coverage is needed to discern the genomes arising from multiple pathogenic strains of the same species that may coexist in the same mixed microbial consortium (23). Considering the current throughput reported by the Illumina NovaSeq 6000 system, this would require at least 1 lane in an S2 flow cell per sample to achieve the needed coverage (Table 1). Therefore, it is more likely to obtain only draft genomes from metagenomic data. Draft genome databases are growing rapidly, and any new microbiological resource deposited in a repository available to the community is announced frequently online in the fully open-access journal *Microbiology Resource Announcements*, published by the American Society for Microbiology. However, many of the draft genomes are contaminated with fragments of sequences from other species (24), and validation of these contigs and draft genomes remains a key essential step (25). However, there is no good validation approach that can assess accuracy in the metagenomic assembly unless a pure culture of that microbial target can be isolated and propagated and whole-genome sequencing is performed, followed by verification against the data derived from metagenomics.

An assembly of metagenomic data would be more useful to elucidate dominant species present in reclaimed water, for example, nitrifying bacteria or heterotrophs that correlate with the nutrient content of the water, because they are more likely to show higher sequencing coverage and, hence, more confident assembly results. However, dominant taxa can be elucidated rapidly using amplicon-based sequencing and may not require the use of metagenomics unless functional annotation is required. Although it is assumed that metagenomics may achieve better resolution and accuracy in taxonomic classifications because more genes associated with the microbial target can be evaluated simultaneously, a recent study suggested the contrary. Tessler et al. analyzed 49 samples from a floodplain system using both 16S rRNA gene-based amplicon and shotgun sequencing (26). Those authors demonstrated that amplicon sequencing could assign more reads at the phylum and family levels and could be relatively more robust across both biodiversity and community ecology analyses than metagenomics. This observation can be explained by the possibility that the taxonomic resolution derived from metagenomics is detrimentally impacted by the coverage and size of whole-genome databases because in instances where whole genomes of target species are absent, many of the reads obtained from shotgun sequencing would be mapped as unknown (26). This error would inherently reduce the number of taxonomically applicable reads. Furthermore, horizontal gene transfer is a ubiquitous and rampant phenomenon in microbial ecosystems (27, 28). Because shotgun sequencing assigns taxonomic classifications based on genes across the entire genome, regardless of whether they are core genes, this can lead to incidences of contradictory and inaccurate identifications if those assigned genes were instead horizontally transferred from another microbial species. In contrast, amplicon sequencing considers only one type of gene at a time and by choosing a core gene (e.g., the 16S rRNA gene), which is rarely transferred horizontally (29), to be sequenced, taxonomical classifications can be assigned more accurately than with metagenomics.

### Availability of bioinformatic tools: gene-centric approach.

Considering the limitations of the genome-centric approach, the alternative gene-centric approach can be used to analyze metagenomic data derived for reclaimed-water quality monitoring. For this approach, the raw reads are input into classifier or profiler programs to map both the phylogenetic and functional profiles of the sample data. For example, interactive toolboxes such as MEGAN (30) provide taxonomic analyses by mapping reads against the NCBI or Silva database. MEGAN also provides functional analysis using various protein databases (e.g., SEED and KEGG). Free public resources such as MG-RAST (31) provide taxonomic and functional analyses similar to those of MEGAN. Additionally, it serves as a public depository for metagenomic data where users interested in metadata analysis can download open-access metagenomic data sets for further data mining. Despite its ease of use, functional analyses with both MG-RAST and MEGAN tend to provide classification of proteins only at the functional class level (e.g., proteins related to biosynthesis, degradation, folding, processing, and modification) (32) and do not facilitate downstream scientific inquiry on the annotated genes that are related to each of these functional classes.

In addition to MEGAN and MG-RAST, in recent years, an increasing number of classification tools (Table 2) have been developed (33). However, the databases associated with each classification method may differ. Some classifiers match DNA sequences obtained from metagenomics to DNA databases, while others match DNA sequences to protein or marker gene databases. To exemplify, common databases include Silva, the Ribosomal Database Project (RDP), Greengenes, and the NCBI database for taxonomic classification or FOAM and PFAM for protein sequences. Depending on these databases, the numbers of taxa or functional genes classified back as output data can differ (34, 35). Ye et al. evaluated the different classifier methods and noted a wide variation in the total species abundances obtained by the different classifiers that have their associated default databases for the same sample. However, if a common database is constructed and used across the different classifier methods, the variation in the total species identified becomes lower (34). Likewise, the antibiotic resistance gene prediction potentials (including the ability to annotate correctly the number of antibiotic resistance genes and associated classes) differ depending on the type of antibiotic resistance database (e.g., ARDB, ARG-miner, CARD, and SARG) (36). These observations are worth noting because companies (e.g., CosmosID, DNAsense, and BaseClear) are now providing metagenomic and bioinformatic services for the generated data, making it particularly convenient for users without any experience handling large data sets to utilize metagenomics as a routine monitoring tool. However, most of these companies use their in-house-developed databases for genome-centric or gene-centric analysis of metagenomic data, making protocol standardization and cross-comparison of results particularly challenging. Therefore, each method or company can provide classification results that differ, which would not facilitate interlaboratory comparisons.

**TABLE 2:** Tools and databases available for phylogenetic identification

Phylogenetic identification tool/database	Version used for this review	Database type	Target collection(s)	Database size (Gb)	Yr of latest update	Time required to build database at first use (h)	Time required to generate classification results^*a*^	Reference
Kraken2	v2.0.8-beta	DNA	RefSeq bacteria	103	2019	15	3 min	66
MiniKraken2	v2	DNA	RefSeq bacteria, archaea, viruses, GRCh38 human genome data set	8	2019	Not required	2 min	66
Kaiju	v1.7.2	Protein	Eukaryotes, bacteria, viral genomes	97	2019	4	2 h	67
MetaPhlAn2	0c3ed7b7718b	Marker gene	Eukaryotes, bacteria, archaea, viruses	1	2018	Not required	2 h	68
mOTUs2	v2.5.1	Marker gene	Eukaryotes, bacteria, archaea	1.5	2018	Not required	40 min	69

a Denotes the time required to generate the classification results from a test data set generated from Illumina HiSeq 4000 paired-end sequencing. The data set contains approximately 7 million trimmed paired reads of an average of 150 bp, with a 600-Mb fastq.gz file.

For some classification methods, particularly those that come with relatively large databases, time is needed to install and build the databases in local servers for first-time users. We performed an analysis to determine the time needed to classify a data set of approximately 890,000 sequences and found that, depending on the method, the time can range from 2 min to 2 h using a one-node CPU and 200 GB of RAM (Table 2). With advances in computing power, the time needed to analyze a full metagenomic data set is likely to shorten. However, there is a likelihood that most of the reads in environmental surveys of reclaimed water can result in being unclassified or unable to be identified confidently at the species/strain level with the profiling methods (37). The collation of large genomic databases remains in its early stages compared with well-curated 16S rRNA gene databases such as RDP, Silva, and Greengenes, particularly for viruses and eukaryotes, for which the completeness of the existing databases may not be as well developed as that for bacteria (38). Furthermore, the classification results derived from shotgun sequencing reads, particularly those that are present at a relative abundance of <0.1%, are likely to represent false-positive identifications (34). Therefore, a bottleneck lies in collating well-curated databases to facilitate our ability to generate meaningful data related to phylogenetic identification from metagenomic data.

In addition to classification for the phylogenetic identities of the microbial community, several databases are available to identify antibiotic resistance genes (ARGs), metal resistance genes, and virulence factors (Table 3). Once the reads are classified accordingly, the mapped reads across a constant can, in theory, be normalized as (i) the number of target sequences per million sequence reads (i.e., counts per million [CPM]), (ii) the number of target sequences per number of marker genes (e.g., the 16S rRNA gene), (iii) the number of target sequences per cell number (39), or (iv) RPKM (reads per kilobase per million mapped reads) (40) or FPKM (fragments per kilobase per million mapped reads) (analogous to RPKM and used especially for paired-end shotgun sequencing reads). CPM is usually more commonly used than RPKM or FPKM. Regardless of the normalization step used, such normalization is required to obtain relative abundances from metagenomics that can be used in comparative analyses. Relative abundances can also be used for correlation against metadata (e.g., water quality data or operational data). For example, Hendriksen and coworkers utilized metagenomics to monitor the occurrence and diversity of ARGs in urban sewage collected from 79 sites in 60 different countries. They expressed the number of reads assigned to ARGs per kilobase per million fragments (FPKM) across the different geographical regions and found that Africa and South America have higher median numbers of ARG reads than Asia, Europe, the Middle East, North America, and Oceania. They further correlated these relative-abundance values with World Bank variables (e.g., extent of open defecation practices, life expectancy, and infection and malnutrition rates) and observed a strong correlation between the relative ARG abundance and socioeconomic, health, and environmental factors (41). This corroborates the conclusion from another study demonstrating a strong correlation between antimicrobial resistance indices (obtained through nonmetagenomic methods) and improving sanitation and good governance (42).

**TABLE 3:** Tools and databases available for marker gene identification^*a*^

Marker gene identification tool/database	Latest version at point of writing	Target collection(s)	Database size	Yr of latest update	Reference
CARD	v3.0.4	ARGs	2,602 genes	2019	70
SARG	v2.0	ARGs	12,307 genes	2018	39
BacMet	v2.0	Antibacterial biocide and metal resistance genes	753 genes (experimentally confirmed); 155,512 genes (predicted)	2018	71
VFDB	Refreshed weekly	VFs	3,220 genes (experimentally confirmed); 28,587 genes (predicted)	2019	72
PAIDB	v2.0	PAIs and REIs	223 PAIs with 1,331 genes; 88 REIs with 108 genes	2015	73
PATRIC	v3.5.43	VFs and ARGs	130,963 VFs; 257,681 ARGs	2019	74
ACLAME	0.4	MGEs	122,154 proteins from 2,326 MGEs	2009	75

a ARGs, antibiotic resistance genes; PAIs, pathogenicity islands; REIs, antimicrobial resistance islands; VFs, virulence factors; MGEs, mobile genetic elements.

Alternatively, multivariate analysis can also be performed using the relative abundances of all identified taxa/genes across the different samples. Changes in the alpha diversity (a quantitative measure of community diversity) of these marker genes identified from metagenomics can also be determined, although there is a need to discern between technical variability (natural changes to a treatment due to the stochastic nature of the system) and biological results (made in response to the treatment) (43). Such an analysis was demonstrated in a recent study that monitored the surface water quality at multiple locations in Haiti postearthquake. The authors of that study determined that the relative abundance of bacteria was differentiated based on the sampling locations, but the Chao1 alpha diversity values were not significantly different among the sampling sites. Those authors further determined the relative abundances of marker genes associated with known waterborne pathogens, such as E. coli O157:H7 and Vibrio cholerae, as well as the presence of phages associated with these pathogens in some of the sample replicates, indicating potential breaches in the sanitation infrastructure after the earthquake (44).

Concerning the genome-centric and gene-centric approaches, see a recent review by Lal Gupta et al., who illustrated a workflow to determine the scope and distribution of resistomes in complex environments using both a read-based profiling approach and a *de novo* assembly-based profiling approach on metagenomic data (36). The workflow suggested by Lal Gupta et al. can potentially be applied to determine the classifications of both taxonomy and other functional genes such as metal resistance genes and virulence factors (Table 3). A large suite of tools for assembly and annotation is available, and each one may generate different results. Choosing the most appropriate or accurate metagenomic tool to facilitate reclaimed-water quality monitoring is not easy because most of the existing tools utilize databases that are not initially developed for this sample type. Several studies were conducted to identify accurate tools for general environmental shotgun sequencing data, with one recent study concluding that k-mer-based approaches (e.g., Kraken) may outperform other tools in terms of accuracy (45) and speed (Table 2). Regardless of which metagenomic analytical pipeline is chosen to be used to determine reclaimed-water quality, the same pipeline should be used consistently across all samples to facilitate comparison.

### Improving pathogen detection capabilities.

In cases of public health outbreaks that may be due to the use of reclaimed water, there is a need to promptly identify the causative microbial agent. However, the current state of metagenomics may not be well poised to facilitate a rapid decision-making process because sample preparation, sequencing, and bioinformatic analysis can consume a considerable amount of time. If the time needed for DNA extraction, library preparation, and sequencing is considered, the whole procedure would have taken approximately 39 to 55 h using an Illumina sequencing platform. This process can be sped up using newer sequencing platforms such as the Nanopore sequencing platforms, but it would still take approximately 18 h to complete the entire preparation and sequencing (46). For example, Nanopore MinION required approximately 24 h to determine the presence of Ebola virus in a human clinical specimen (47). However, viral genomes are small (<1 Mbp) and do not represent the average genome size of bacterial or protozoal pathogens. Hence, the time needed to draft a complete genome of large prokaryotic or eukaryotic cells would be significantly longer. Alternatively, instead of focusing on complete genomes, the draft genomes of bacterial isolates can be obtained through metagenomics. The reads are merged and assembled to obtain longer contigs or draft genomes before mapping against bacterial pathogen databases. The contigs can then be identified for marker genes associated with pathogenic species at a certain threshold confidence level. Using this approach, bacterial pathogens such as Bacillus anthracis, Klebsiella pneumoniae, and nontuberculous mycobacteria were detected in the effluent of a wastewater treatment plant (WWTP) that utilized only a conventional activated sludge tank to decontaminate the wastewater (48). This approach can be further sped up to provide a preliminary analysis of the functional traits within 6 h of sequencing on the Nanopore platforms (46).

Although metagenomics has demonstrated the huge potential to reveal novel insights into gene functions, identifying pathogens through assembly may be challenging because waterborne pathogens are generally present in low abundances and would theoretically show up with very low read counts. For example, with approximately 4 Gb per library, pathogenic E. coli isolates that tested positive using culture-based methods were not detected by metagenomics (49). Brute-force ultradeep sequencing can be performed to obtain a high read coverage of those rare taxa, but this approach can be costly. In recent years, within the field of clinical diagnostics, attempts have been made to identify pathogens using a scoring system after metagenomics. To do so, sequences are obtained from both background controls and test samples before alignment and identification against a curated database (e.g., nucleotide or protein databases of the NCBI). The number of reads that aligned positively to a known hit (e.g., target X) in the database is determined first in the background/control samples. This would generate a mean number of reads assigned to target X along with the standard deviation that is present in the background/control samples. Subsequently, the number of reads assigned to target X in a separate test sample can also be obtained. A Z-score can then be obtained (50), and one can then denote which target demonstrates the highest Z-score regardless of its raw abundance and, hence, presumably is the causative agent of a clinical infection. This or similar scoring approaches have been tested for clinical diagnostics, where samples are derived from blood, urine, or biopsy specimens (50–53). However, no demonstration of this approach has yet been made on reclaimed-water samples because it may be technically challenging to do so given the more diverse microbial community in reclaimed water than in infected clinical specimens.

### Improving semiquantitative capabilities of metagenomics.

Most of the studies expressed marker genes as a relative abundance, calculated using the following equation:

$$\text{relative abundance of marker gene }x=\frac{\text{(number of sequences assigned to gene }x\text{)}}{\text{(total number of sequences)}}$$

However, this calculation does not consider the reference sequence length and how it would impact match hits (54). For example, in the SNC-ARDB database for ARGs, reference sequences can range from 186 to 4,728 bp. The number of reads signifying marker gene *x* that map positively to a reference sequence of 186 bp may be different from the number of reads that map positively to a reference sequence of 4,728 bp when using the same criteria of ≥90% sequence identity and an alignment length of ≥25 amino acids. Hence, Li et al. demonstrated a correction factor that normalizes the number of sequences assigned to gene *x* by the reference sequence length (54). In the same study, they further expressed the reads in a way similar to those obtained from quantitative PCR, whereby they normalized the marker gene *x* results to the total number of reads that matched against the 16S rRNA reference sequence, using the following formula:

$$\begin{array}{c} \text{relative abundance of marker gene }x\text{ normalized against 16S rRNA genes}=\frac{\text{(number of sequences assigned to gene }x\text{)(length of reads/length of gene }x\text{ reference sequence)}}{\text{(number of sequences assigned to 16S)(length of reads/length of 16S reference sequence)}} \end{array}$$

Alternatively, metagenomic data can also be assessed for the average coverage of a set of approximately 30 essential single-copy marker genes that were found in nearly all *Bacteria* and *Archaea* (39, 55). Because these are single-copy marker genes, the average number of these genes can be interpreted to be similar to the numbers of bacterial and archaeal cells. Subsequently, this value can be used as a normalization factor to determine the number of reads of marker gene *x* per prokaryote cell.

The above-mentioned methods used for metagenomic data sets can achieve information on relative abundance only and cannot provide quantitative measurements in terms of the number of contaminants per liter of reclaimed water. The latter set of values is usually needed for quantitative microbial risk assessment (QMRA). A possible way to overcome this challenge would be to couple flow cytometry with metagenomics for the same sample. For example, the total cell counts can be first estimated by enumerating them using nucleic acid stains and flow cytometry. This would generate a value associated with the number of cells per liter. This value can then be multiplied by the normalized marker gene *x* count per prokaryote cell obtained via metagenomics to derive the marker gene *x* count per liter. However, even with these estimated values, dose-response models and transmission probabilities associated with emerging contaminants such as antibiotic-resistant bacteria (ARB) or ARGs are still unavailable to facilitate QMRA, although recent efforts have been made to introduce dose-response models that incorporate stochastic death dynamics between ARB and antibiotic-susceptible bacteria (56), hence allowing the consideration of ARB in existing dose-response models.

### Applications of metagenomics to monitor reclaimed-water quality.

Metagenomics is commonly used to conduct a baseline characterization of the diversity and relative abundance of contaminants that are present in reclaimed water. For example, Chopyk et al. collected water samples from tidal brackish rivers, freshwater ponds and creeks, and water reclamation facilities and proceeded to process these samples for shotgun metagenomics (57). The samples were evaluated for taxonomic and functional differences. Although no apparent differences were found in the overall phylogenetic distributions of the microbial communities among the samples, the diversity of ARGs in at least one of the reclaimed-water samples was greater than that in the other water samples. This outlier trend may be an anomaly arising from the small sample size or a potential breach in the treatment process.

In addition to ARGs, the diversity of viruses that are present in reclaimed water can also be elucidated by metagenomics. Most of the assigned reads obtained from metagenomics were determined to be bacteriophages assigned to the families *Myoviridae*, *Podoviridae*, and *Siphoviridae* (58–60). In contrast, human enteric viruses account for <1% of the total sequences obtained from treated effluent after membrane filtration (58). By matching against databases designed to annotate viral sequences (e.g., MetaVir), viruses of potential public health relevance and belonging to the families *Herpesvirales*, *Adenoviridae*, *Polyomaviridae*, and *Parvoviridae* are detectable in the post-membrane-filtrated effluents (58). Coincidentally, *Polyomaviridae* viruses were also detected in the post-membrane-filtrated chlorinated effluent sampled from a WWTP at another location (60). These earlier studies use a gene-centric approach to identify the marker genes associated with potential viruses at the family level and, hence, cannot describe the viral pathogens at the species level. Additionally, most of the detected human enteric viruses are double-stranded DNA viruses and not single-stranded RNA viruses that would need to first be recovered through RNA extraction and transcribed to obtain cDNA before proceeding with shotgun metagenomic sequencing.

The above-mentioned studies characterized microbial contaminants that are present in reclaimed water collected at the end of the wastewater treatment process. This sampling point is typically defined as the point of entry before the reclaimed water is transported or distributed to the point of use. Because reclaimed water typically still contains organic carbon and other essential nutrients that can support microbial regrowth, the reclaimed-water quality can potentially change within the distribution network depending on factors such as the residual disinfectant concentration, hydraulic retention time, distance of the network, and so on. To determine changes in water quality and, hence, infer the extent of biological stability of reclaimed waters, metagenomics can be used to characterize the microbial community in the reclaimed water at the point of use and compare it against that at the point of entry. Garner et al. determined that in four of their studied reclaimed-water distribution networks, decreases were observed in the relative abundances and diversities of ARGs from the point of entry to the point of use. However, the relative abundances of certain ARGs correlate with the concentration of biological dissolved organic carbon, suggesting the need to limit the amount of organic carbon in distribution systems (61). Similarly, Zaouri et al. utilized a metagenomic approach to simultaneously monitor the taxonomic profiles of bacterial and viral communities as well as the antibiotic resistome in aquifers that were recharged with treated wastewater (62). The authors determined that bacterial families such as *Planctomycetes* are present at a high relative abundance in recharged aquifers compared to the upstream controls, likely because of the higher organic carbon content in these waters upon exposure to treated wastewater. This observation reiterates the above-mentioned observation that organic carbon can change the microbial community, likely because of microbial regrowth. Additionally, Zaouri et al. observed that the viral family *Picornaviridae* is present at a high relative abundance in recharged aquifers compared with the controls (62), suggesting the potential dissemination of human enteric viruses at the point of use due to reclaimed water.

Collectively, these studies demonstrate the use of metagenomics to (i) identify microbial populations and functional genes in water matrices, (ii) compare samples for reclaimed-water quality on either a temporal or a spatial scale, and (iii) correlate data from metagenomics to other metadata (e.g., organic content, residual disinfectants, and temperature) to determine which variable to control to alleviate unwanted detrimental changes in reclaimed-water quality.

### Perspectives.

Metagenomics provides a nontargeted approach to simultaneously examine both phylogenetic and functional profiles associated with water matrices. However, to revamp the way in which the water industry is monitoring reclaimed-water quality, continued development in metagenomics is needed in the following areas:

**(i) Improving databases.** There should be a continuous effort to perform whole-genome sequencing of a wide consortium of biological pathogens relevant to reclaimed water, particularly viruses and protozoa. These assembled genomes should be made available in public depositories for further curation of databases, which would improve the resolution of future information that we can obtain from metagenomics.

**(ii) Standardized protocols for data analysis.** Similar to other methods that are endorsed by regulatory agencies for water quality monitoring, shotgun sequencing protocols and bioinformatic pipelines should also be standardized so that metagenomic data can be benchmarked against regulatory standards and cross-compared across different laboratories.

**(iii) Developing bioinformatic tools to identify rare taxa (e.g., low-abundance pathogens).** While brute-force ultradeep sequencing can help in identifying rare taxa, this incurs a cost that can add up significantly if routinely adopted for reclaimed-water quality monitoring. The huge amount of data would also need more time for analysis to be completed. To circumvent this bottleneck, rapid bioinformatic tools need to be developed to identify low-abundance pathogens and samples with poor water quality. Potential tools include the Z-scoring system already demonstrated for clinical samples, which would need to be fine-tuned for reclaimed-water quality monitoring, and data mining or a machine-learning algorithm to identify trends and outliers that can isolate aberrations in reclaimed-water quality.

**(iv) Conducting more studies to demonstrate the use of metagenomics for reclaimed-water quality monitoring.** Developing metagenomics as a toolkit to denote and predict water quality would require more studies to provide a representative sample size that can identify which biomarkers correlate with certain measurable water quality data (e.g., pH, residual chlorine, and organic carbon concentration). Current studies mainly focus on monitoring reclaimed water for the microbial community and ARGs. Other functional genes, such as mobile genetic elements, virulence factors, and metal resistance genes, also play a role equal to that of ARGs in affecting potential safety concerns when reusing waters and should also be evaluated in future studies.

### Conclusions.

The advent of next-generation sequencing technologies and faster computing capabilities and the availability of databases have facilitated the use of metagenomics for reclaimed-water quality monitoring. Metagenomics can determine changes in both the phylogenetic and functional diversities of emerging contaminants in a nontargeted manner. Such information can be used to elucidate the removal efficiency achieved by wastewater treatment technologies and to monitor changes in reclaimed-water quality over a distribution network. The data derived from metagenomics are semiquantitative (i.e., in terms of relative abundance). However, when complemented with other tools, for example, flow cytometry and quantitative PCR, estimated abundance data can be derived, although more studies are required to facilitate the use of these data in risk assessment or for comparison against regulatory limits.
